# Prognostic impact of dynamic changes of type I melanoma antigen gene proteins CT7 (*MAGE-C1/CT7*) transcripts in multiple myeloma

**DOI:** 10.3389/fmed.2025.1566265

**Published:** 2025-05-09

**Authors:** Xuelin Dou, Fengrong Wang, Huan Chen, Yao Chen, Lei Wen, Yang Liu, Guorui Ruan, Xiaosu Zhao, Xiaojun Huang, Robert Peter Gale, Jin Lu

**Affiliations:** ^1^Peking University People’s Hospital, Peking University Institute of Hematology, National Clinical Research Center for Hematologic Disease, Beijing Key Laboratory of Hematopoietic Stem Cell Transplantation, Peking University, Beijing, China; ^2^Centre for Haematology, Department of Immunology and Inflammation, Imperial College of Science, Technology and Medicine, London, United Kingdom; ^3^Collaborative Innovation Center of Hematology, Soochow University, Suzhou, China

**Keywords:** multiple myeloma, prognostic biomarker, *MAGE-C1/CT7*, cancer testis antigen genes, novel agents

## Abstract

Type I melanoma antigen gene proteins CT7 (*MAGE-C1/CT7*), a cancer-testis (CT) gene, correlated with clinical parameters at diagnosis of multiple myeloma (MM). We first analyzed single-cell ribonucleic acid sequencing data from public databases to evaluate the expression of *MAGE-C1/CT7* in MM patients and showed that *MAGE-C1/CT7* is highly and specifically expressed in the MM cells. We then interrogated data from 216 consecutive cases with *MAGE-C1/CT7* transcripts by quantitative real-time polymerase chain reaction longitudinally monitored in our center. The positive rate of *MAGE-C1/CT7* at baseline was 87.3%, with a median level of 4.46% (0.01–939.5). In univariate Cox regression analysis, peri-ASCT *MAGE-C1/CT7* status showed better discriminatory ability in PFS and survival than peri-ASCT multi-parameter flow-cytometry status assessed by flow cytometry. In multivariate analysis, patients who were *MAGE-C1/CT7-*negative pre-transplant and posttransplant had significantly better PFS than those who were positive in both determinations (HR = 0.33, 95% CI: 0.14, 0.80, *p* = 0.01). In 69 patients with informative samples, we found a 2-log decrease in *MAGE-C1/CT7* transcript concentration after the second induction cycle correlated with achieving negative *MAGE-C1/CT7*-test results both pre-transplant and posttransplant (OR = 6.08, 95% CI: 1.78, 20.74, *p* = 0.004). Our data showed the predictive value of peri-ASCT frontline treatment. A 2-log decrease of *MAGE-C1/CT7* post-induction cycle 2 compared to baseline correlated with a negative peri-ASCT *MAGE-C1/CT7* status, providing an earlier prognostic marker of treatment response.

## 1 Introduction

The advent of novel agents, including proteasome inhibitors (PIs), immunomodulatory agents (IMiDs), and monoclonal antibodies (mAbs), has brought unprecedented improvement in the treatment of multiple myeloma (MM), with autologous stem-cell transplantation (ASCT) remaining as a crucial part (1–3). The prognostic value of measurable residual disease (MRD) status, determined by either next-generation flow (NGF) or next-generation sequencing (NGS) under such modern treatment modality, has been reconfirmed in various studies (4). However, the issues of cost, accessibility, and applicability of those measures should not be neglected.

The concentration of transcripts of the type I melanoma antigen gene proteins CT7 (*MAGE-C1/CT7*), a cancer-testis gene, at diagnosis, is reported to correlate with clinical co-variates, including extent of bone marrow plasma cell infiltration, cytogenetic abnormalities, and PFS (5–7). We wondered whether dynamic changes in *MAGE-C1/CT7* transcript concentrations would correlate with outcomes in patients receiving novel agents and autologous hematopoietic cell transplant (ASCT) as initial therapy.

In this study, we first evaluated the expression of *MAGE-C1/CT7* in MM patients by analyzing the public single-cell ribonucleic acid (RNA) sequencing (scRNA-seq) datasets. We then retrospectively enrolled 216 MM patients treated with novel agents as induction therapy followed by autologous stem cell transplantation (ASCT) at our center over the past 10 years, with longitudinal monitoring of *MAGE-C1/CT7* levels in the bone marrow. Our aim is to investigate the role of dynamic changes in *MAGE-C1/CT7* as a predictor of outcomes and to provide insights into the efficacy and clinical outcomes of these patients.

## 2 Methods

### 2.1 Subjects

Medical records of 260 patients with multiple myeloma receiving induction therapy, including a proteasome inhibitor and an ASCT as initial therapy at Peking University People’s Hospital from March 2011 to November 2021, were reviewed. There were 216 patients with *MAGE-C1/CT7* transcript concentration testing included. Patients received 4–6 cycles of triplet regimens, including bortezomib, dexamethasone with or without thalidomide, lenalidomide, or daratumumab, followed by an ASCT with high-dose melphalan as induction. Tandem ASCT was recommended in patients with ≥2 of the following: (1) del(17p); (2) t (4;14); (3) t (14;16); (4) t (14;20); (5) 1q gain or amplification; and (6) extramedullary disease with extraosseous lesions. Posttransplant maintenance therapy was based on risk stratification, drug accessibility, and affordability. Patients with standard-risk cytogenetics received lenalidomide or thalidomide, or if contradicted, daratumumab. Patients with high-risk cytogenetics, defined as the presence of del17p, t (4;14), or t (14;16) (8), received both a proteasome inhibitor and an IMiD. The study was approved by the Ethics Committee of Peking University People’s Hospital, and patients gave written informed consent compliant with the precepts of the Helsinki Declaration.

### 2.2 Definition and monitoring

Patients diagnosed before 2015 meet the International 2003 Myeloma Working Group (IMWG) diagnostic criteria and those thereafter the 2014 IMWG diagnostic criteria for multiple myeloma (9, 10). Responses were assessed according to the IMWG response criteria (11). The extramedullary disease was defined as para-skeletal soft-tissue masses, soft-tissue masses spreading outside the bone marrow, or both. The last follow-up time was January 2023. Endpoints included progression-free survival (PFS) and survival.

### 2.3 Multi-parameter flow-cytometry (MPFC), fluorescent *in situ* hybridization (FISH), and quantitative real-time polymerase chain reaction (qRT-PCR)

A bone marrow aspirate was obtained pre-therapy, after the second cycle of induction therapy, within 30 days pretransplant, and 100 d (± 30 d) posttransplant. MRD was assessed using an 8-color antibody panel of CD38/CD138/CD45/CD19/CD56/CD117/cytoplasmic kappa (cκ)/cytoplasmic lambda (cλ). Patients receiving daratumumab <3 months before MPFC testing had an additional panel of CD38/CD229/CD45/CD19/CD56/CD117/cκ/cλ. Sensitivity was 10E-4 to 10E-5. FISH was done pretherapy using CD138-positive plasma cells processed by magnetic-activated cell sorting (MACS) as described (12). Samples were analyzed for 1q21+, del(17p), del(13q), and *IGH* rearrangement using gene locus-specific probes (GLPs), including GLP 1q21, GLP P53, GLP D13S391, GLP RB1, and GLP *IGH*. If an *IGH* rearrangement was identified, dual-color and dual-fusion translocation probes such as IgH-FGFR3, IgH-MAF, and *IGH*-CCND1 were used to detect t(4;14)(p16;q32), t(14;16)(q32;q23), and/or t(11;14)(q13;q32). Three (gain) or ≥4 (amp) copies of 1q21 were combined and termed 1q21-postive. High-risk cytogenetic abnormalities were defined by the presence of del(17p), t(4;14), or t(14;16).

*MAGE-C1/CT7* transcript concentrations were determined more than once by qRT-PCR. A 10-μL PCR mixture contained 5 μL 1 × TaqMan® Universal PCR Master Mix (Applied Biosystems, Foster City, California), 400 nmol/L primers, 250 nmol/L fluorescent probes, and 150–500 ng cDNA. PCR was performed with the ABI PRISM® 7500 FAST Sequence Detection System (Applied Biosystems, Waltham, MA). *MAGE-C1/CT7* transcript concentration was quantified by qRT-PCR using *ABL1* as an internal control. Primers and probes were as follows: *ABL* (forward 5’-CCGCTGACCATCAATAAGGAA-3,’ reverse 5’-GATGTAGTTGCTTGGGACCCA-3,’ and probe 5’-FAM-CCATTTTTGGTTTGGGCTTCACACCATT-TAMARA-3′); *MAGE-C1/CT7* (forward 5’-TTGTCTTCTGGGAACCTTGACTC-3,’ reverse 5’-TGAGGGACACATACATCCTAAAAGC-3,’ and probe 5’-FAM-ACTGCCTGGGCCTCCTCTGCTGT-BHQ-3′). Quantification was done against the *ABL1* standard curve to decrease experimental error. Sensitivity was 1–10 copies in the plasmid DNA standards and 10^−4^–10^−5^ in bone marrow specimens (13).

### 2.4 Analysis of single-cell RNA sequencing datasets

The scRNA-seq datasets were downloaded from the National Center for Biotechnology Information (NCBI)‘s Gene Expression Omnibus with accession numbers GSE234261 and GSE161195. For scRNA-seq analysis, the R package Seurat (v.4.1.1)^1^ was used (14). First, to perform quality control, various filters were applied to the data to exclude barcodes falling into any one of these categories: too few genes expressed (possibly debris) or too many unique molecular identifiers (UMIs) associated (possibly more than one cell). Next, the data were normalized and scaled, and dimensional reduction was performed using principal component analysis (PCA). Finally, the expression levels of *MAGE-C1/CT7* in the MM microenvironment and CD138 positive MM cells of MM patients were visualized.

### 2.5 Statistics

Descriptive statistics were used to summarize co-variates. The χ2 or Fisher exact tests were used for categorical co-variates and a non-parametric test for continuous co-variates. Receiver operating characteristic (ROC) curve and time-dependent ROC curves were analyzed using the timeROC package in the R platform (15). Cutoff values were determined with the largest Youden index for PFS defined above. The area under the ROC (AUROC) was used to estimate the accuracy of the predictive model. Survival functions were estimated using the Kaplan–Meier method and compared using the log-rank test. PFS and survival were defined as the interval from the ASCT to progression, recurrence, or death in the PFS model and death from any cause in the survival model. The concordance index (C-statistic) was also calculated to assess and compare the prognostic ability of pre-transplant and posttransplant *MAGE-C1/CT7* and MPFC status for progression-free survival. The C-statistic represents the probability that, for a randomly selected pair of patients with different PFS times, the model correctly predicts which patient progressed earlier. Cox proportional hazards regression models were used to evaluate co-variates associated with PFS and survival. Univariable Cox regression analyses for co-variates associated with PFS and survival included sex (M/F), age (continuous), estimated glomerular filtration rate (eGFR; < *vs*. ≥40 mL/min), extramedullary disease (extramedullary bone disease *vs*. N; extraosseous disease *vs*. N), ISS (II *vs*. I, III *vs*. I), R-ISS (II *vs*. I, III *vs*. I) and R2-ISS (II *vs*. I, III *vs*. I, IV *vs*. I); ASCT conditioning intensity (100–140 *vs*. 200 mg/mE+2); complete response at day +100 (Y/N); posttransplant maintenance therapy (Y/N); pre-transplant and posttransplant MPFC state (MPFC ^+/− or −/+^ *vs*. MPFC ^++^, MPFC ^−−^ *vs*. MPFC ^++^); and *MAGE-C1/CT7* state (*MAGE-C1/CT7* ^+/− or −/+^ *vs*. *MAGE-C1/CT7* ^++^, *MAGE-C1/CT7* ^−−^ *v*s. *MAGE-C1/CT7* ^++^). *p*-values are two-sided. There was an interaction between ISS, R-ISS, and R2-ISS prognostic staging systems. The most accurate was selected for multivariate analysis by the highest C-statistic. Co-variates with *p* < 0.2 in univariable analyses were included in multivariable analyses and selected using a backward elimination process to fit a Cox regression model. Co-variates with *p* < 0.05 were considered significant. Statistical analyses and graphing were done with R version 4.2.1 (R Core Team, Vienna, Austria) and SPSS 26.0 software (SPSS, Chicago, IL).

## 3 Results

### 3.1 *MAGE-C1/CT7* is highly expressed in the MM cells

**Figure 1:**
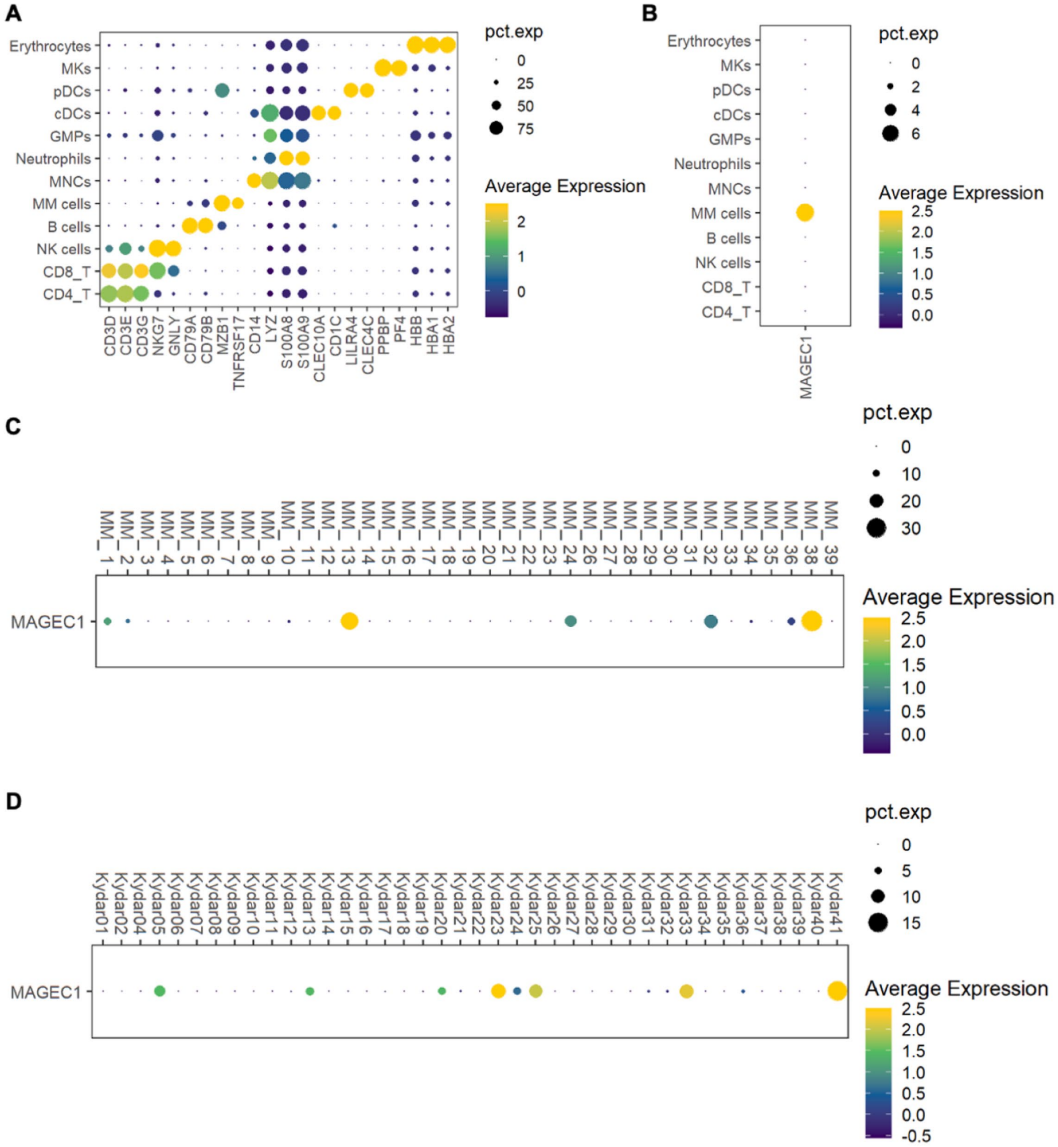
Delineation of the expression of *MAGE-C1/CT7* in patients’ MM microenvironment and MM cells. **(A)** Dot plot of the representative marker genes for each cell type within the MM microenvironment. **(B)** Dot plot showing the expression of *MAGE-C1/CT7* in each cell type. **(C)** Dot plot displaying the *MAGE-C1/CT7* expression in the MM cells of each patient from public dataset GSE234261. **(D)** Dot plot depicting the expression of *MAGE-C1/CT7* in the MM cells of each patient from public dataset GSE161195. cDCs, conventional dendritic cells; GMPs, granulocyte-monocyte progenitors; MKs, megakaryocytes; MM cells, multiple myeloma cells; MNCs, monocytes; NK cells, natural killer cells; pDCs, plasmacytoid dendritic cells; pct.exp., the percentage of cells expressing the given gene.

To investigate *MAGE-C1/CT7* expression in MM patients, we analyzed a public single-cell RNA sequencing (scRNA-seq) dataset (GSE234261), which includes bone marrow (BM) samples from 39 MM patients. Following quality control (Supplementary Figures S1A,B), we classified the retained cells into 12 types based on marker gene expression profiles, including MM cells (MZB1 and BCMA), T cells (CD4 + and CD8+, marked by CD3D, CD3E, and CD3G), NK cells (NKG7 and GNLY), B cells (CD79A and CD79B), monocytes (CD14 and LYZ), neutrophils (S100A8 and S100A9), granulocyte-macrophage progenitors (GMPs and LYZ), conventional dendritic cells (cDCs, CLEC10A, and CD1C), plasmacytoid dendritic cells (pDCs, LIRA4, and CLEC4C), megakaryocytes (MKs, PPBP, and PF4), and erythrocytes (HBB, HBA1, and HBA2) (Figure 1A). MM cells displayed elevated *MAGE-C1/CT7* expression compared to other microenvironmental cells (Figure 1B). Further analysis identified five patients (12.8%) with high *MAGE-C1/CT7* expression in MM cells (Figure 1C). To validate these findings, we analyzed another public dataset (GSE161195) containing scRNA-seq data of CD138 + MM cells from 40 patients (Supplementary Figures S2A,B). This dataset showed seven patients (17.1%) with high *MAGE-C1/CT7* expression (Figure 1D). Together, our results indicate that *MAGE-C1/CT7* is highly and specifically expressed in MM cells.

### 3.2 Patient co-variates and therapy

Data from 260 patients receiving novel agents-based induction therapy and a frontline ASCT as consolidation therapy at Peking University People’s Hospital from March 2011 to November 2021 were included in the study. Thirty-one cases without *MAGE-C1/CT7* testing were excluded. Baseline co-variates of the included 216 patients are displayed in Table 1, with 121 (56%) being men. Median age was 54 years (Interquartile range [IQR], 48–60 years). Forty-seven (22%) had extramedullary disease. Forty-seven (22%) cases had high-risk cytogenetics, including del(17p) (*N* = 11), t (4;14) (*N* = 38), or t (14;16) (*N* = 1). Eighty-four (39%) cases had 1q21 gain or amplification. International Scoring System (ISS) staging, revised ISS (R-ISS) staging, and Second Revision of the ISS (R2-ISS) staging are displayed in (Supplementary Figure S3).

**Table 1:** Baseline subject co-variates.

Co-variates	Total (*N* = 216)	Baseline *MAGE-C1/CT7* expression			*p*-value^&^
		Negative (*N* = 20)	Positive (*N* = 137)	Missing (*N* = 59)	
Median age y (range)	54 (27, 69)	53 (39, 64)	55(27, 69)	51 (33, 68)	0.47
Male, *n* (%)	121 (56%)	14 (70%)	77 (56%)	30 (51%)	0.24
M-protein *N*. (%)					0.09
IgG	104 (48%)	9 (45%)	70 (51%)	25 (42%)	
IgA	46 (21%)	8 (40%)	20 (15%)	18 (31%)	
IgD	12 (6%)	0 (0%)	9 (7%)	3 (5%)	
Bi-clonal (IgG + IgA)	4 (2%)	0 (0%)	4 (3%)	0 (0%)	
Kappa light-chain	20 (9%)	1 (5%)	15 (11%)	4 (7%)	
Lambda light-chain	26 (12%)	1 (5%)	17 (12%)	8 (14%)	
Non-secretory	4 (2%)	1 (5%)	2 (2%)	1 (2%)	
Creatinine clearance at diagnosis < 40 mL/min, *n* (%)	15 (7%)	1 (5%)	13 (10%)	1 (2%)	0.51
LDH > upper limit of normal, *n* (%)	29 (13%)	1 (5%)	20 (14%)	8 (14%)	0.24
Bone marrow plasma cells < 10%, *n* (%)	25 (12%)	9 (45%)	12 (9%)	4 (7%)	<0.001
Extramedullary disease	47 (22%)	11 (55%)	26 (19%)	10 (17%)	< 0.001
del(17p), *n* (%)					0.59
Yes	11 (5%)	1 (5%)	9 (7%)	1 (2%)	
No	181 (84%)	16 (80%)	125 (91%)	40 (68%)	
Missing	24 (11%)	3 (15%)	3 (2%)	18 (31%)	
t (4;14), *n* (%)					
Yes	38 (18%)	7 (35%)	24 (18%)	7 (12%)	0.09
No	145 (67%)	10 (50%)	106 (77%)	29 (49%)	
Missing	33 (15%)	3 (15%)	7 (5%)	23 (39%)	
t (14;16), *n* (%)					
Yes	1 (0.5%)	0 (0%)	1 (0.7%)	0 (0%)	0.82
No	182 (84%)	17 (85%)	129 (94%)	36 (61%)	
Missing	33 (15%)	3 (15%)	7 (5%)	23 (39%)	
t (11;14), *n* (%)					
Yes	35 (16%)	4 (20%)	27 (20%)	4 (7%)	0.38
No	148 (69%)	13 (65%)	103 (75%)	32 (54%)	
Missing	33 (15%)	3 (15%)	7 (5%)	23 (39%)	
Cytogenetic risk, *n* (%)					0.16
Standard	137 (63%)	10 (50%)	98 (72%)	29 (49%)	
High risk*	47 (22%)	7 (35%)	33 (24%)	7 (12%)	
Missing	32 (15%)	3 (15%)	6 (4%)	23 (39%)	
1q21+^^^, *n* (%)					0.13
Yes	84 (39%)	5 (25%)	65 (47%)	14 (24%)	
No	108 (50%)	12 (60%)	69 (50%)	27 (46%)	
Missing	24 (11%)	3 (15%)	3 (2%)	18 (31%)	
ISS stage, *n* (%)					0.42
I	65 (30%)	10 (50%)	43 (31%)	12 (20%)	
II	82 (38%)	6 (30%)	53 (39%)	23 (39%)	
III	66 (31%)	4 (20%)	40 (29%)	22 (37%)	
Missing	3 (1%)	0 (0%)	1 (0.7%)	2 (3%)	
R-ISS stage, *n* (%)					0.52
I	52 (24%)	8 (40%)	37 (27%)	7 (12%)	
II	115 (53%)	10 (50%)	71 (52%)	34 (58%)	
III	35 (16%)	2 (10%)	26 (19%)	7 (12%)	
Missing	14 (7%)	0 (0%)	3 (2%)	11 (19%)	
R2-ISS stage, *n* (%)					0.26
I	40 (19%)	7 (35%)	25 (18%)	8 (14%)	
II	53 (25%)	4 (20%)	34 (25%)	15 (25%)	
III	95 (44%)	8 (40%)	55 (40%)	32 (54%)	
IV	25 (12%)	1 (5%)	22 (16%)	2 (3%)	
Missing	3 (1%)	0 (0%)	1 (0.7%)	2 (3%)	

^*^High-risk defined as del(17p), t (4;14), or t (14;16).

^&p-value denotes the comparison between the negative and positive MAGE-C1/CT7 expression cohorts at baseline. Subjects with missing values were excluded.^

^$^Bone, other, or none.

^^^Three (gain) or ≥4 (amp) copies of 1q21 were combined and designated 1q21 + .

CR, complete response; LDH, lactate dehydrogenase; ISS, International Scoring System; R-ISS, revised ISS; R2-ISS, Second Revision of the ISS.

For induction therapy, all patients received triplets, including bortezomib and dexamethasone. One hundred and eighteen (55%) also received thalidomide, lenalidomide, and/or pomalidomide. Sixteen (7%) received daratumumab. Others received chemotherapy, including cyclophosphamide and adriamycin. The median interval from induction therapy start to transplant was 8 months (IQR, 6.5–9.4 months). One hundred and seventy-three (80%) received induction with a melphalan dose of 200 mg/m^2^, and 43 (20%) received 100–140 mg/m^2^. Seventeen patients (8%) received tandem ASCT. Of 203 patients (94%) receiving maintenance therapy, 97 (48%) received lenalidomide, 55 (27%) thalidomide, 20 (10%) bortezomib, 4 (2%) daratumumab, and 28 (14%) both a proteasome inhibitor and an IMiD.

### 3.3 Correlation of *MAGE-C1/CT7* transcript concentration with baseline co-variates

Patient baseline co-variates by *MAGE-C1/CT7* transcript concentration are displayed in Table 1. Quantification of bone marrow *MAGE-C1/CT7* transcript against the *ABL1* expression level was positive in 137 (87%) patients, with a mean fluorescence intensity of 4.5% (0.01–939.5), and was negative in 20 (13%) (Supplementary Figure S4). *MAGE-C1/CT7* was not tested in the other 59 patients at baseline. Frequencies of patients with bone marrow plasma cell percentage <10% in the *MAGE-C1/CT7* transcript-positive and -negative cohorts were 45 and 9% (*p < 0.001*). Frequencies of extramedullary disease also differed, 19% *vs.* 55% (*p < 0.001*). No significant differences were observed between the cohorts in age, sex, M-protein type, creatinine clearance, lactate dehydrogenase (LDH), cytogenetic risk cohort, 1q21 abnormality, and ISS, R-ISS, and R2-ISS stages.

### 3.4 Response and survival

The overall response rate (ORR) was 94% (95% confidence interval [CI], 91–97%) pretransplant, including 96 patients (43%) with a complete response (CR), 55 (26%) with a very good partial response (VGPR), and 52 (24%) with a partial response (PR). At day 100 posttransplant, 143 (66%) patients were in CR, 39 (18%) in VGPR, and 17 (8%) in PR.

The median duration of follow-up is 41 months (IQR, 23–56 months). There was progression or death in 77 patients (36%); 44 patients (20%) died, including 1 at <100 d posttransplant from infection. Median durations of PFS and survival were 79 months (95% CI, 67–114 months) and 123 months (95% CI, 107–NE months); 2-, 4-, and 6-year PFSs were 76% (95% CI, 70–82%), 61% (95% CI, 54–70%), and 54% (95% CI, 45–65%). Corresponding survivals were 92% (95% CI, 89–96%), 79% (95% CI, 73–86%), and 70% (95% CI, 62–80%).

### 3.5 Co-variates associated with PFS and survival

**Figure 2:**
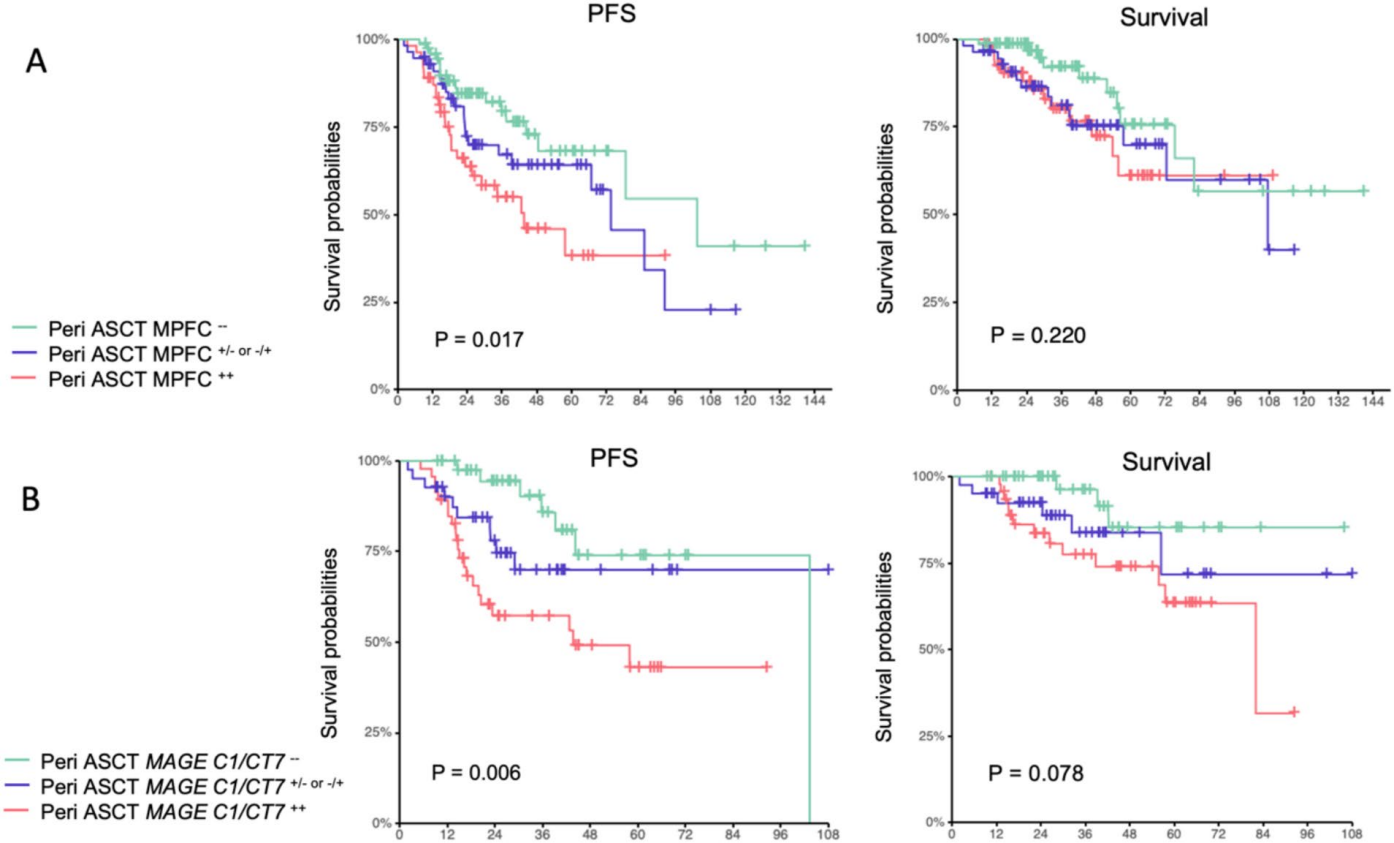
Progression-free survival and survival of pre-transplant and posttransplant MPFC state **(A)** and pre-transplant and posttransplant MAGE-C1/CT7 transcripts **(B)**.

We first interrogated the correlations between pre-transplant and posttransplant MPFC and *MAGE-C1/CT7* concentrations and posttransplant outcomes. Pre-transplant and posttransplant MPFC data were available for 181 patients (84%), *MAGE-C1/CT7* concentration data in 130 patients (60%), and both for 127 patients (59%). In 84 patients (66%), the results of pre-transplant and posttransplant MPFC and *MAGE-C1/CT7* concentrations were concordant (Supplementary Figure S5). All patients were divided into three cohorts: (1) MPFC-positive pre-transplant and posttransplant (MPFC^++^; *N* = 55); (2) MPFC-positive at either pre-transplant or posttransplant (MPFC^+- or −+^; *N* = 56); and (3) MPFC-negative pre-transplant and posttransplant (MPFC^−−^; *N* = 70). Similarly, patients were divided into: (1) *MAGE C1/CT7*-positive pre-transplant and posttransplant (*MAGE-C1/CT7*^++^, *N* = 47); (2) *MAGE C1/CT7*-positive pre-transplant or posttransplant (*MAGE-C1/CT7*^+/−or−/+^, *N* = 41); and (3) *MAGE C1/CT7*-negative pre-transplant and posttransplant (*N* = 42). The Kaplan–Meier plots of PFS and survival for these cohorts are displayed in Figure 2. Pre-transplant and posttransplant *MAGE-C1/CT7* concentrations (C-statistic = 0.75 [0.65, 0.85]) showed a trend toward being a more accurate predictor of PFS compared to MPFC data (C-statistic = 0.65 [0.55, 0.75], *p* = 0.12). Neither pre-transplant nor posttransplant *MAGE-C1/CT7* transcript concentration nor MPFC-test results were significantly correlated with survival.

**Figure 3:**
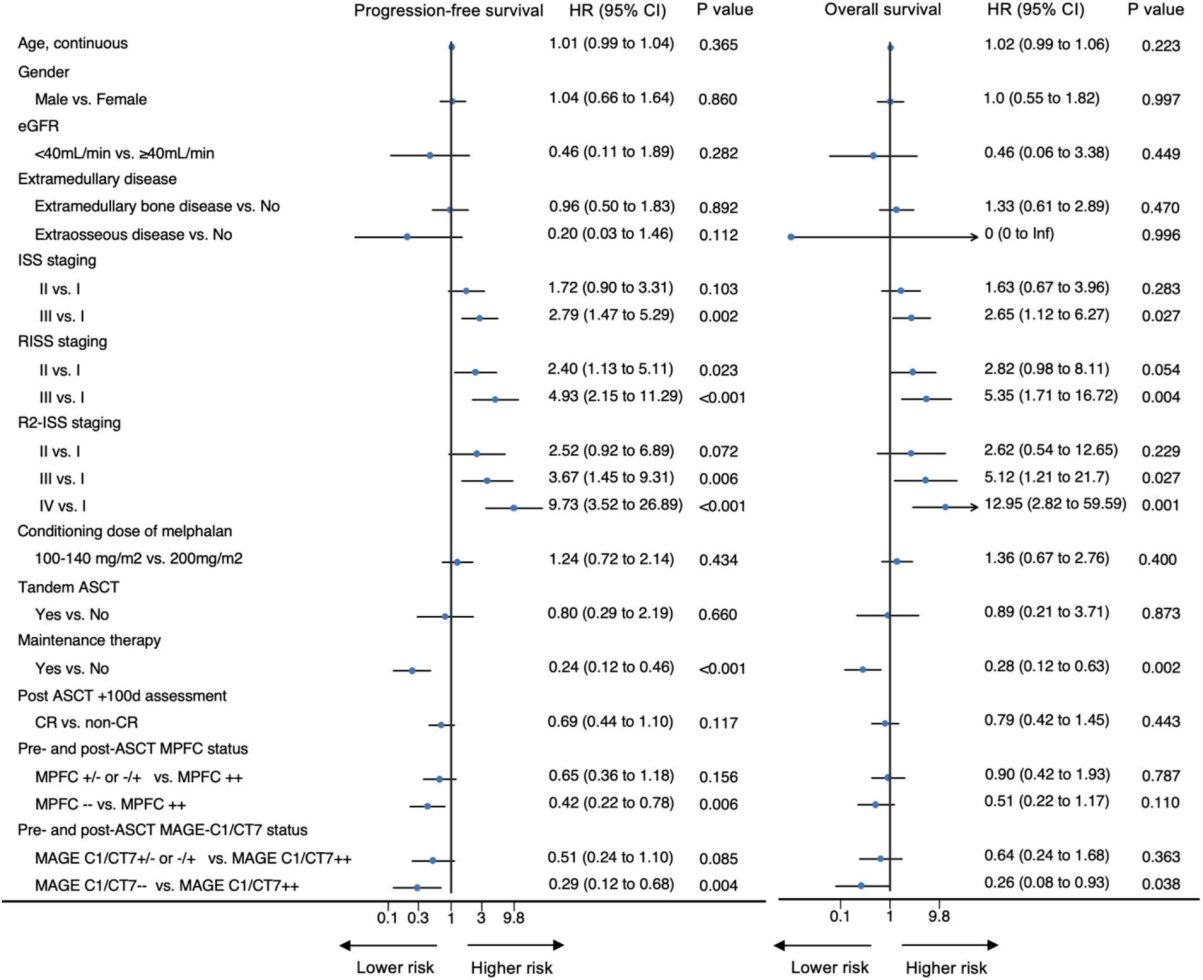
Univariable Cox regression analyses of co-variates for PFS and survival.

The results of univariable analyses are displayed in Figure 3. R2-ISS stage was used in multivariable analyses because it provided better survival prediction compared to ISS or R-ISS (Supplementary Figures S6, S7). Other covariates included in the multivariable analyses of PFS and survival included response on day +100 (PFS), maintenance therapy, pre-transplant or posttransplant MPFC state, and *MAGE-C1/CT7* transcript concentration. The results are displayed in Table 2. R2-ISS staging (II *vs.* I; hazard ratio [HR] = 2.32 [0.50, 10.82]; *p* = 0.29; III *vs*. I; HR = 4.23 [0.97, 18.46]; *p* = 0.06; IV *vs*. I; HR = 6.07 [1.15, 31.96]; *p* = 0.03; overall *p* = 0.09) and pretransplant *MAGE-C1/CT7* concentration (*MAGE-C1/CT7*^+/−or−/+^ *vs*. *MAGE-C1/CT7*^++^; HR = 0.64 [0.29, 1.42]; *p* = 0.27; MAGE-C1/CT7^−−^ vs. *MAGE-C1/CT7*^++^; HR = 0.33 [0.14, 0.80]; *p* = 0.01; overall *p* = 0.05) were significantly associated with PFS, but not survival. Maintenance therapy was significantly associated with PFS (HR = 0.29 [0.09, 0.97]; *p* = 0.04) and survival (HR = 0.13 [0.05, 0.37]; *p* < 0.001).

**Table 2:** Multivariable Cox regression analyses of co-variates associated with PFS and survival.

Co-variates	PFS		Survival	
	HR (95% CI)	*p*	HR (95% CI)	*p*-value
R2-ISS staging		0.09		
II vs. I (ref.)	2.32 (0.50–10.82)	0.29		
III vs. I (ref.)	4.23 (0.97–18.46)	0.06		
IV vs. I (ref.)	6.07 (1.15–31.96)	0.03		
Posttransplant maintenance therapy (Yes vs. No)	0.29 (0.09–0.97)	0.044	0.13 (0.05–0.37)	<0.001
Complete response at day 100 posttransplant (Y *vs*. N)	0.76 (0.48–1.20)	0.24		
Pre-transplant and posttransplant MAGE-C1/CT7 state		0.045		
MAGE C1/CT7^+/− or −/+^ *v*s. MAGE C1/CT7^++^(ref.)	0.64 (0.29–1.42)	0.27		
MAGE C1/CT7^−−^ *vs*. MAGE C1/CT7^++^ (ref.)	0.33 (0.14–0.80)	0.014		

PFS, progression-free survival. R2-ISS, Second Revision of the International Scoring System.

### 3.6 Correlation between *MAGE-C1/CT7* dynamics during induction and pre-transplant and posttransplant *MAGE-C1/CT7* concentration

We examined correlations between *MAGE-C1/CT7* transcript concentration dynamics and pre-transplant and posttransplant *MAGE-C1/CT7* transcript concentration. Sixty-nine patients had analyzable samples across the four time points. Median *MAGE-C1/CT7* transcript value was 18% (0.01–532%) of the *ABL1* control. The kinetics of reduction of *MAGE-C1/CT7* transcript concentration was calculated with the value after the second induction course divided by the value at baseline. Using AUROC curves, we determined the optimal quotient predicting a pre-transplant and posttransplant *MAGE-C1/CT7*-negative state as 0.008, with an AUROC of 0.77 (0.64, 0.91; *p* < 0.001). Thus, an undetectable level or a > 2-log decrease after the second induction course was determined to be the optimal cutoff value. Thirty-eight patients met this criterion after the second induction course compared to baseline, and among them, 18 (47%) became pre-transplant and posttransplant *MAGE C1/CT7-*negative. In contrast, only 4 of the 31 patients (13%) without a > 2-log decrease became pre-transplant and posttransplant *MAGE C1/CT*7-negative (odds ratio [OR] = 6.08 [1.78–20.74]; *p* = 0.004).

## 4 Discussion

We found patients who were *MAGE-C1/CT7-*positive pre-transplant and posttransplant had significantly worse PFS compared to those who were negative at both time points. Moreover, in patients with informative samples, we found a 2-log decrease in *MAGE-C1/CT7* transcript concentration after the second induction cycle correlated with achieving a negative *MAGE-C1/CT7*-test result pre-transplant and posttransplant. These data indicate that analyzing *MAGE-C1/CT7-*transcript concentration correlates with PFS but does not result in survival in patients with multiple myeloma receiving triplet induction therapy including a proteasome inhibitor and an ASCT as initial therapy. We suggest that the lack of a correlation with survival reflects the impact of post-progression interventions.

Cancer-testis antigens (CTAs) are a class of tumor-associated antigens normally expressed in immune-privileged tissues such as germ cells of the testis and, in some instances, the trophoblast of the placenta. Their expression is typically silenced in somatic tissues (16). The aberrant re-expression of CTAs such as *MAGE-C1/CT7* in various cancers, including multiple myeloma, makes them potential targets for immunotherapeutic interventions due to their restricted expression profile in normal tissues and their immunogenicity (6). Our initial analysis of single-cell RNA sequencing data from public databases demonstrated that *MAGE-C1/CT7* is highly and specifically expressed in MM cells compared to other microenvironmental cells. We observed elevated *MAGE-C1/CT7* expression in MM cells compared to other cell types, including B cells, T cells, NK cells, monocytes, and erythrocytes. Given that *MAGE-C1/CT7* is a cancer-testis antigen typically silenced in somatic tissues, its observed high expression in myeloma cells suggests a relative specificity. However, it should be noted that the public datasets utilized for our initial analysis did not include data on normal plasma cells, necessitating further investigation to definitively assess *MAGE-C1/CT7* expression in their normal counterpart. Previous studies have frequently reported the expression of CTAs, including *MAGE-C1/CT7* as one of the most common, in bone marrow samples from patients with multiple myeloma (6, 17). *MAGE-C1/CT7* has been implicated in promoting myeloma progression and has been explored for diagnostic purposes (5, 7, 18–20). *MAGE-C1/CT7* has been shown to play a role in myeloma cell survival by inhibiting apoptosis and influencing cell cycle regulation (17, 21). Its expression has also been linked to poorer prognosis and early relapse (22). In pilot studies, bone marrow expression of *MAGE-C1/CT7* correlated with clinical co-variates, including the extent of bone marrow plasma cell infiltration, cytogenetic abnormalities, and relapse risk (5, 13, 23). However, these studies were small, had brief follow-ups, did not consider other prognostic or predictive co-variates, and were done before the use of current therapies. To address some of these limitations and to further understand the specificity of *MAGE-C1/CT7* expression in the context of myeloma, our study initially utilized publicly available scRNA-seq datasets to confirm the expression profile of *MAGE-C1/CT7* in myeloma cells.

BM MRD detection measures, such as NGF and NGS, allow for deepened assessment of treatment responses, and their independent prognostic role has been reconfirmed in various prospective clinical trials in MM (4, 24, 25). However, both NGF and NGS require established facility access as NGS was performed on the EuroFlow™ platform and NGS in cross-validated laboratories with authorized assay (26). NGF also requires higher specimen volume, and NGS needs a baseline patient sample for future identification and detection (27). The expression of BM *MAGE-C1/CT7* had a lower cost and lesser demand for facility access, providing an alternative MRD monitoring measure for regions with limited resources.

The emergence of BM MRD detection methodologies with a sensitivity reaching 10^−6^, i.e., NGF and NGS, allowed for deepened assessment of treatment responses after achieving CR and were widely used as a surrogate endpoint as their unequivocally independent prognostic role had been reconfirmed in various prospective clinical trials (4, 25). However, a considerable number of discordant results existed between the achievement of CR and MRD negativity. Bruno Paiva et al. (28) had made a thorough discussion of the possible explanation and concluded that the probability of a false-positive detection of M-proteins is greater than a false-negative MRD test. On this opinion, we would like to advocate and reemphasize that M-proteins or light chains were indirect measurements of tumor burden since they were only secreted by MM cells, whereas BM MRD detection was a more direct and specific way to quantitate tumor burden. In this manner, *MAGE-C1/CT7* should also be considered as a proper candidate for MRD measurement.

Taking the risk factors of 1q21 and the combination of multiple chromosomal abnormalities into consideration, Mattia D’Agostino et al. (29) showed that the R2-ISS staging system allowed a better stratification of MM patients collecting data from different trials. R2-ISS staging system had been validated in other cohorts (30, 31), yet not in cohorts with MM patients receiving novel agents and ASCT as frontline therapy only. In this specific group of MM patients, our data also substantiated the more robust prognostic role of R2-ISS staging than ISS and R-ISS staging.

Our study is limited by its retrospective nature, potentially introducing biases. Furthermore, complete *MAGE-C1/CT7* expression levels across four time points were unavailable for all patients. This was primarily due to the evolving implementation of this biomarker testing during the study period and limitations in the availability of original diagnostic samples for retrospective analysis. Thus, the association of peri-ASCT *MAGE-C1/CT7* status with survival outcomes was assessed first, followed by evaluating the correlation between the change after the second induction cycle and peri-ASCT *MAGE-C1/CT7* status. Additionally, *MAGE-C1/CT7* was not unanimously expressed in the BM of bone marrow patients as only 87.3% of patients showed positive value at diagnosis, indicating that a part of patients could not be tracked by this gene. However, it should be noted that other MRD methods also had limitations; for example, NGS could also only be tracked in approximately 90% of patients. While our study utilized MPFC for MRD assessment, we acknowledge its lower sensitivity (10^−4^ to 10^−5^) compared to more advanced techniques such as NGS, which can reach a sensitivity of 10^−6^. However, our study focused on evaluating the clinical utility of *MAGE-C1/CT7* transcript quantification, which, in our hands, demonstrated a comparable sensitivity of 10^−4^ - 10^−5^ to MPFC. Importantly, *MAGE-C1/CT7* testing offers potential advantages in terms of accessibility and cost-effectiveness, particularly in resource-limited settings where NGF/NGS may not be readily available. Therefore, while acknowledging the limitations of MPFC sensitivity, our findings suggest that *MAGE-C1/CT7* could serve as a valuable and more accessible biomarker for prognostication and treatment response monitoring in MM. Finally, the absence of an independent validation cohort also restricts the generalizability of our findings. Ideally, prospective studies with an independent cohort comparing the sensitivity and specificity of dynamic change in *MAGE-C1/CT7* with NGF or NGS in predicting outcomes should be carried out to further validate our conclusion.

## 5 Conclusion

Our data indicate an independent PFS predictive value of *MAGE-C1/CT7* testing in patients with multiple myeloma receiving novel therapy and ASCT as frontline treatment. A 2-log decrease of *MAGE-C1/CT7* post-induction cycle 2 compared to baseline correlated with negative *MAGE C1/CT7* status pre- and post-ASCT, providing an earlier indication for prognosis.

## Data Availability

The data analyzed in this study is subject to the following licenses/restrictions: data will be made available upon reasonable request. Requests to access these datasets should be directed to lujin@pku.edu.cn.

## Footnotes

^1^ http://satijalab.org/seurat/
