# Supplementary material for: Prognostic impact of dynamic changes of type I melanoma antigen gene proteins CT7 (MAGE-C1/CT7) transcripts in multiple myeloma
Source: Front Med (Lausanne). 2025 May 9;12:1566265. doi: 10.3389/fmed.2025.1566265 (PMC12098646; doi:10.3389/fmed.2025.1566265)
Supplement: Supplementary file 1 [file Data_Sheet_1.docx]

**Supplemental Data**

| **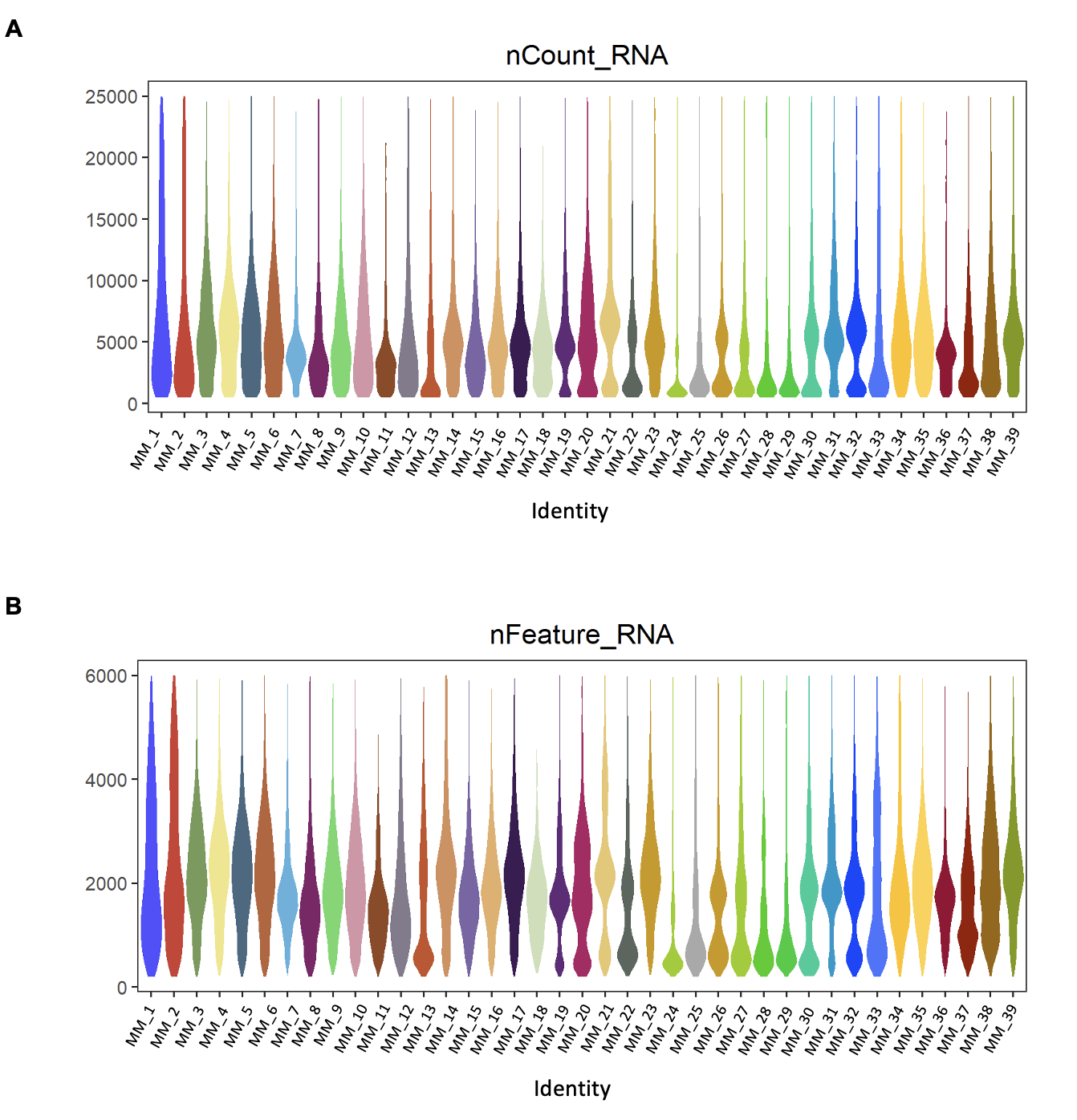** |
| --- |
| **Supplementary Figure S1.** Quality control of the scRNA-seq dataset. Violin plots showing the number of total RNAs (A) and the number of genes (B) for cells from 39 specimens (GSE234261). |

| **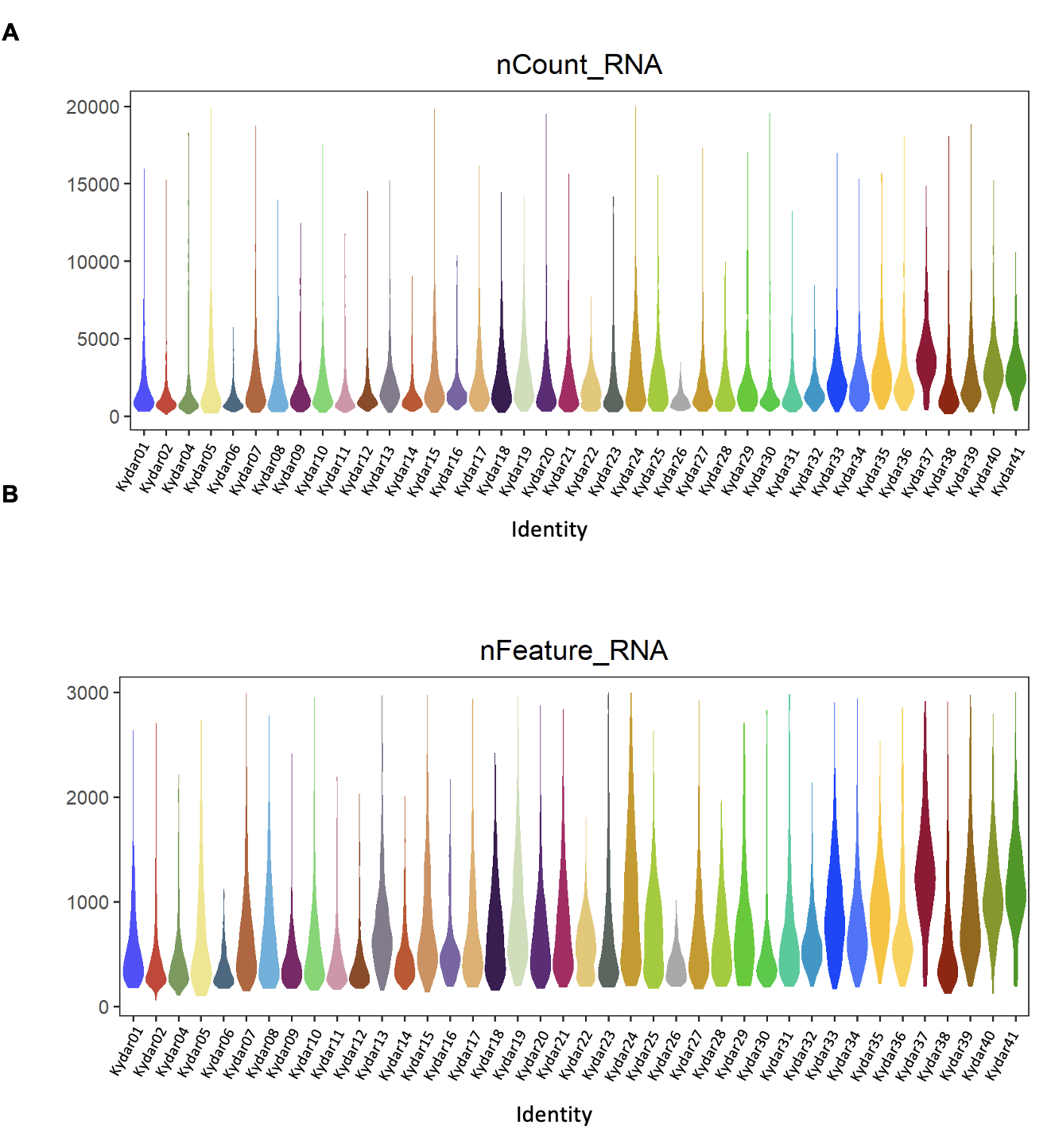** |
| --- |
| **Supplementary Figure S2.** Quality control of the scRNA-seq dataset. Violin plots showing the number of total RNAs (A) and the number of genes (B) for cells from 40 specimens (GSE161195). |

**
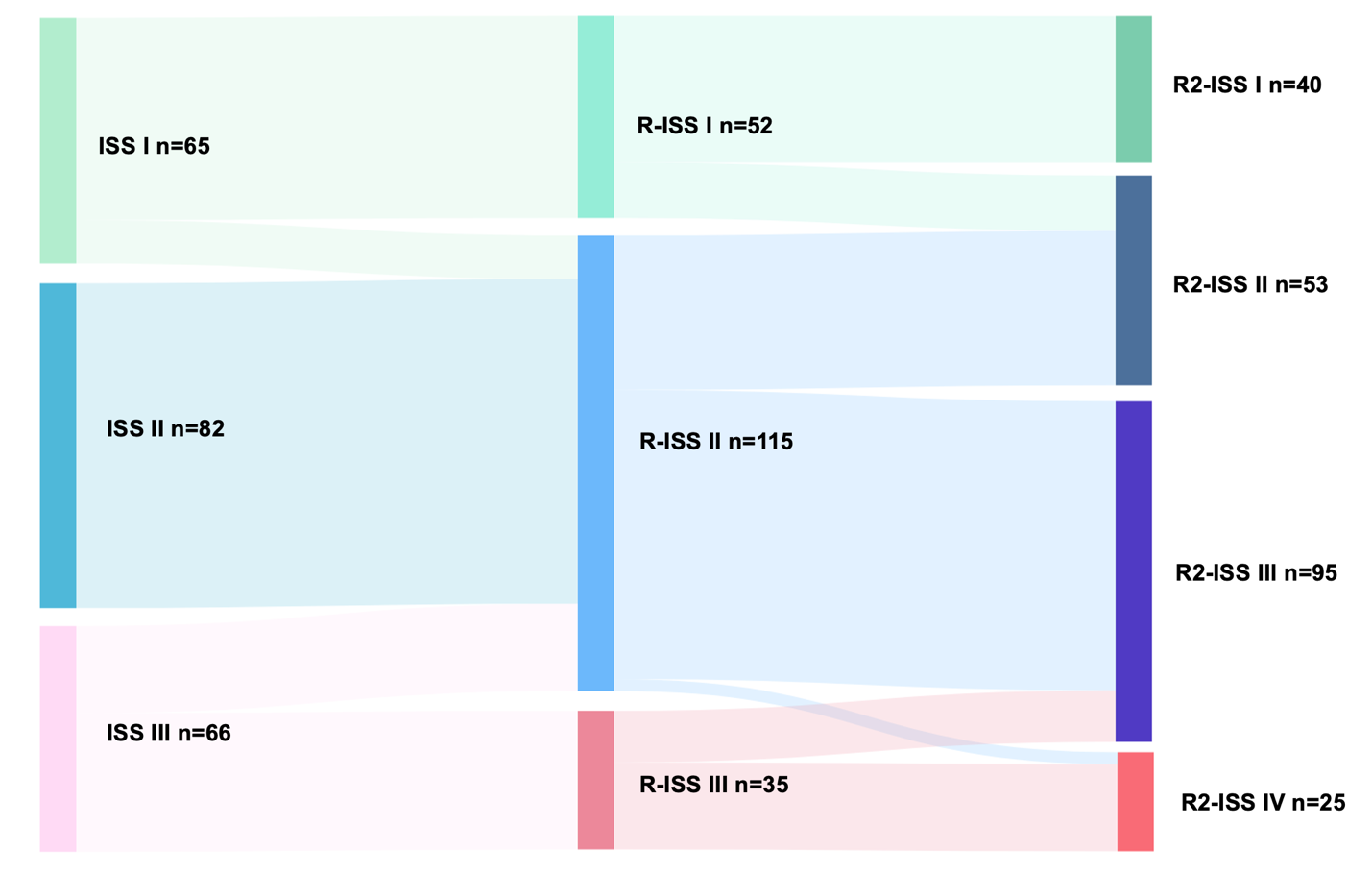
**

**Supplementary Figure S3.** Flow of staging systems**.** Abbreviations: ISS, International Scoring System (ISS) staging; R-ISS, revised ISS staging; R2-ISS, Second Revision of the ISS.

**
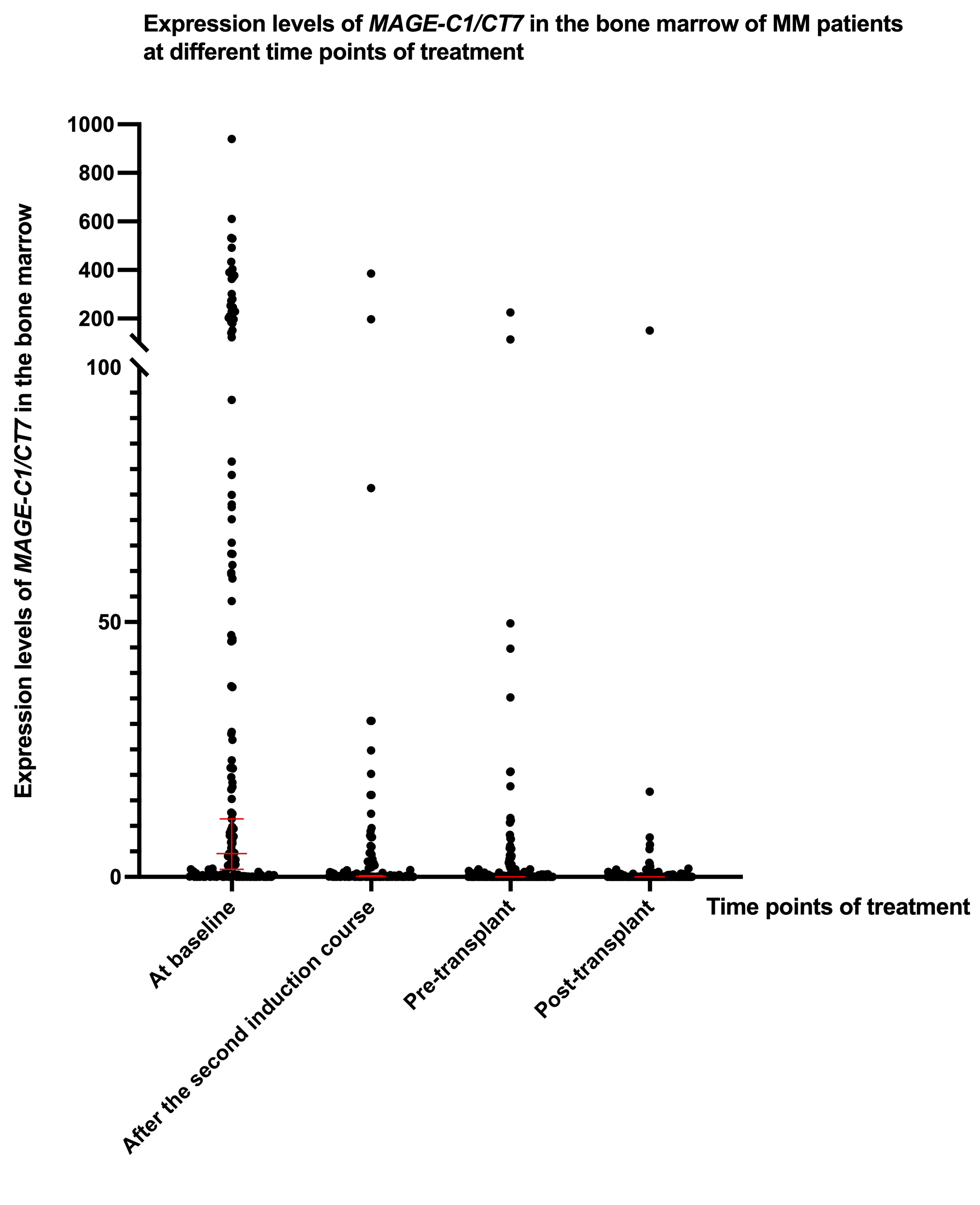
**

**Supplementary Figure S4.** Expression levels of MAGE-C1/CT7 in the bone marrow of MM patients at different time points of treatment. Red line denotes median value with 95% confidence interval.

**
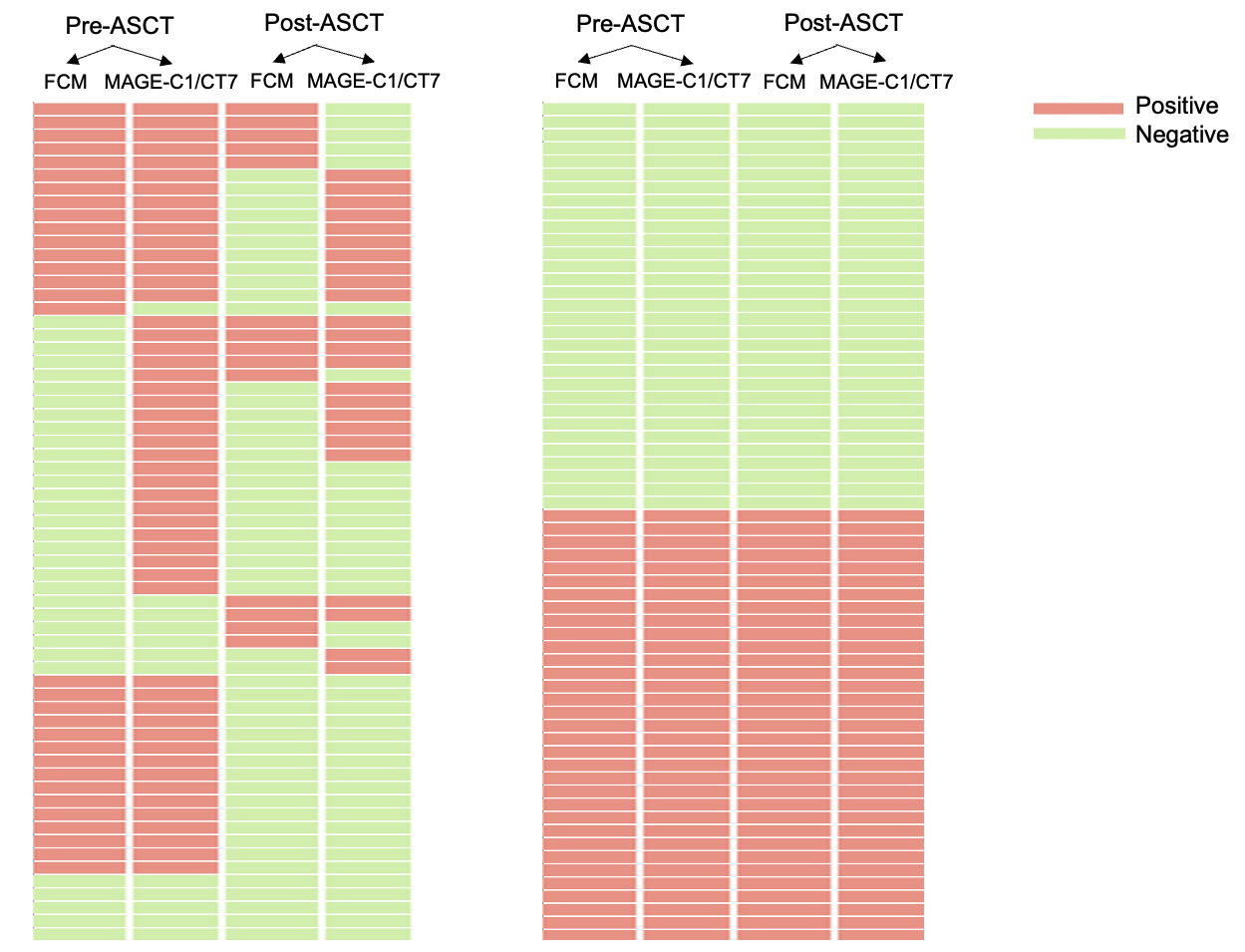
**

**Supplementary Figure S5.** Pre- and posttransplant MPFC and *MAGE-C1/CT7* state in 127 subjects. In 84 (66%) results of pre- and posttransplant MPFC and *MAGE-C1/CT7* transcript concentration were concordant.


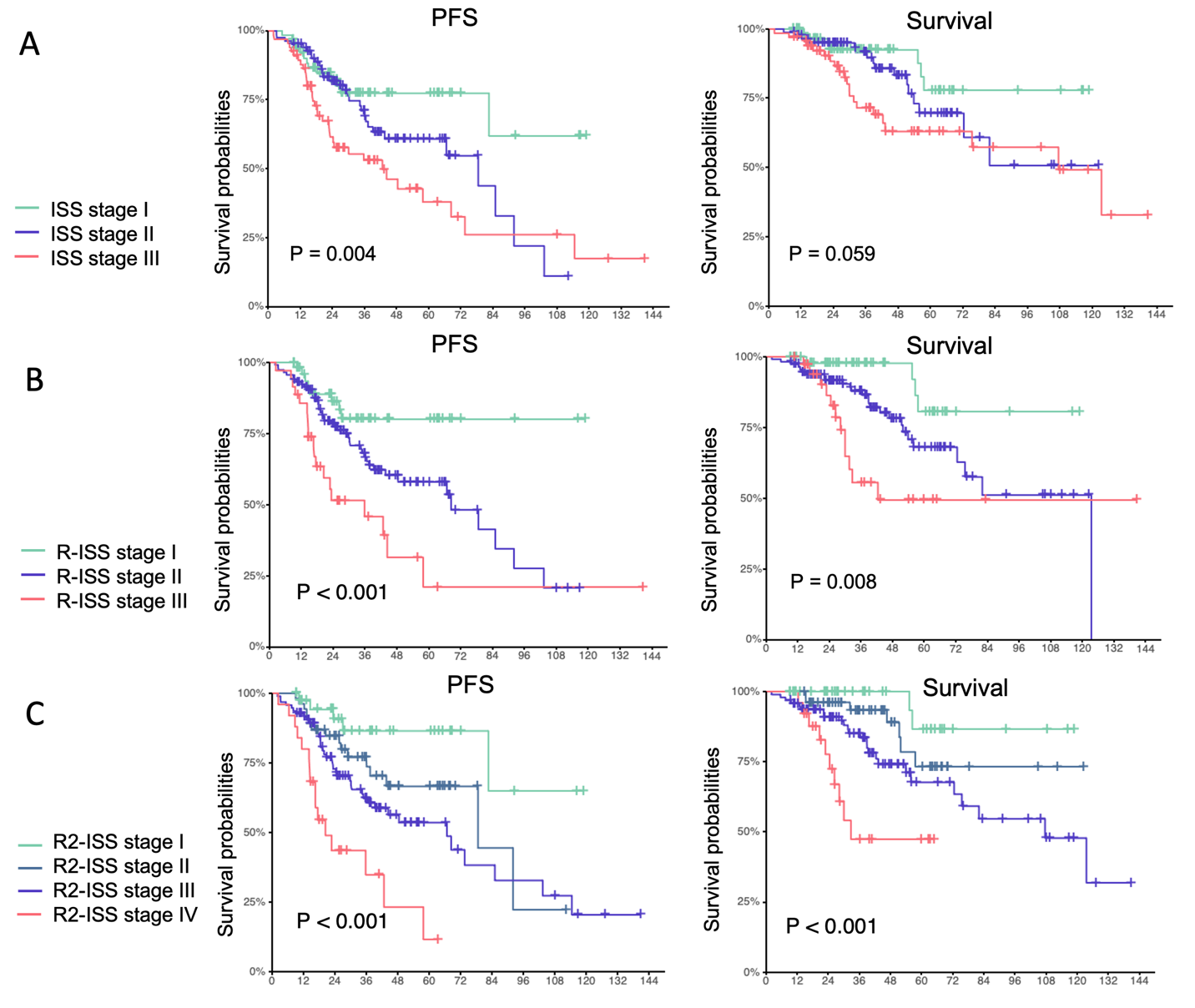


**Supplementary Figure S6.** PFS and survival by ISS (A), R-ISS (B) and R2-ISS (C) stage.

**
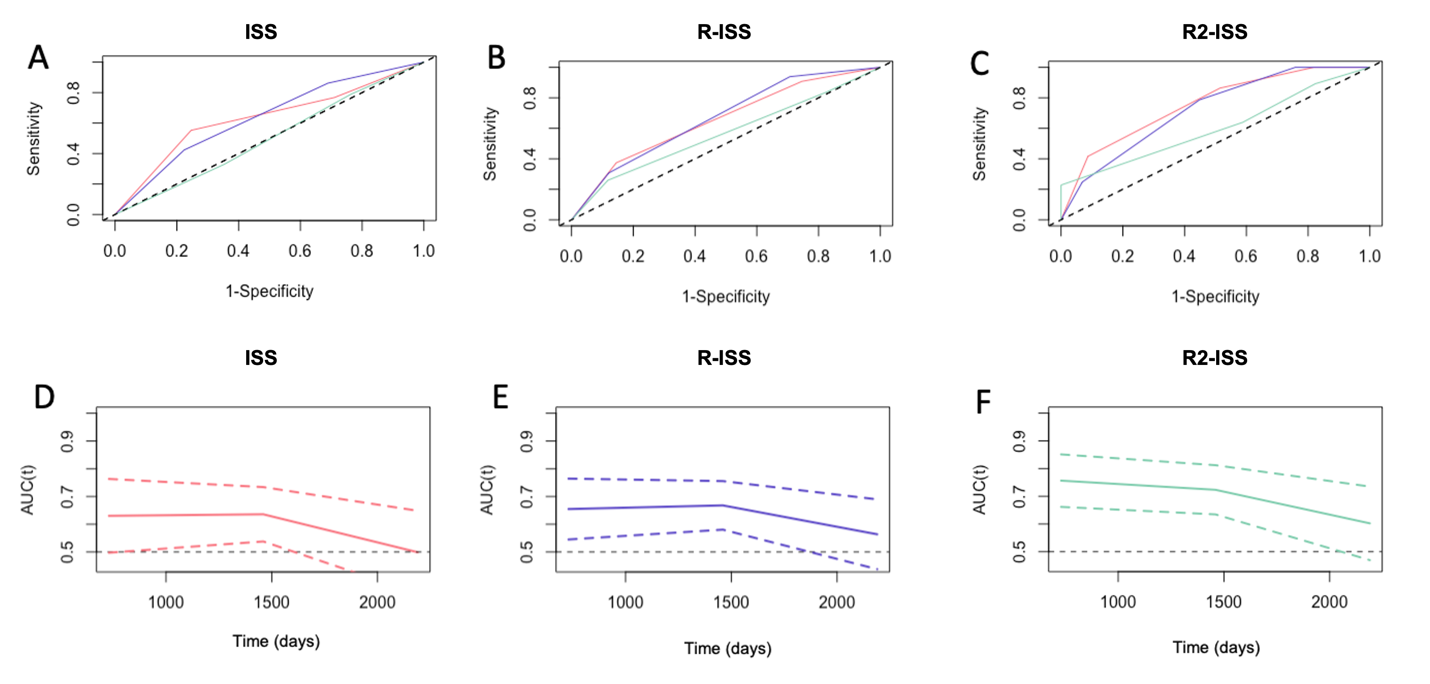
**

**Supplementary Figure S7.** AUROC curves for PFS at 2-, 4-, and 6-years for the ISS (A), R-ISS (B) and R2-ISS (C) staging system and the time-dependent AUROC curves for the ISS (D), R-ISS (E) and R2-ISS (F) staging system. R2-ISS staging showed better sensitivity and specificity with a C-statistic of 0.76 (0.66, 0.85) for 2-year PFS, 0.72 (0.63, 0.81) for 4-year PFS and 0.60 (0.47, 0.74) for 6-year.
